# Eco-friendly approach to improve traits of winter wheat by combining cold plasma treatments and carbonization of subtropical biomass waste

**DOI:** 10.1038/s41598-022-15286-4

**Published:** 2022-07-02

**Authors:** Mahin Saberi, Hamid Ghomi, Christian Andreasen

**Affiliations:** 1 Department of Agricultural Science, Tarbiat Modares University, Tehran, Iran; 2 Department of Plant and Environmental Sciences, University of Copenhagen, Taastrup, Denmark; 3 Laser and Plasma Research Institute, Shahid Beheshti University, Tehran, Iran

**Keywords:** Biophysics, Plant sciences

## Abstract

This study aims to improve the quality and quantity of winter wheat by using the potential of combining the use of cold plasma and waste biorefinery products for improving wheat yield. Plasma was applied by a radio frequency (RF) plasma reactor operated with air for 180 s and 50 W. The waste biorefinery products, including pyroligneous acid, biochar, and azolla compost, were used as plant nutrition. The effects of cold plasma treatment and waste biorefinery products were determined by measuring plant photosynthesis, grain yield, and content of chlorophyll, carotenoids, anthocyanin, protein, and starch. The experiment was conducted during the cropping seasons 2016−18 in a randomized complete block design with four replications. The combination of cold plasma and pyroligneous acid increased the grain yield up to 40.0%. The photosynthesis rate was improved up to 39.3%, and total chlorophyll content up to 48.3% in both years. Seed plasma treatment combined with biochar application increased the starch content by 36.8%. Adding azolla compost increased the protein content by 35.4%. Using seed plasma treatment with biochar increased the microbial biomass carbon by 16.0%. The application of plasma and azolla compost increased the microbial biomass nitrogen by 29.0%.

## Introduction

Climate change and population growth require an increase in agricultural production per unit area, and healthy crops and food safety play an essential role in the human health community^1^. Although the excessive application of chemicals in agriculture may increase the yield per unit area, it could cause problems concerning the quality and safety of agricultural products^2^. According to Balfour (2006), “the human physiological well-being and spiritual has roots in the soil,” and also, “you are what you eat”, indeed refers to the relationship between dietary composition and human physiology^3^. Therefore, in a sustainable agricultural system, besides yield, proper nutrient cycling management and long-term soil fertility should be taken into consideration^4^.

Recently, atmospheric-pressure cold plasma has been considered as a novel technique to improve grain yield and seed germination^5,6^. Plasma is one of the four fundamental states of matter and was first described by chemist Irving Langmuir as an ionized gas containing different oxygen radicals, charged particles, ions, and UV light^7,8^. Hempedu Bumi (*Andrographis paniculata* (Burm. f.) Wall. ex Nees) seeds, treated with a Dielectric Barrier Discharge (DBD) at 5950 V for 10 s, had faster germination and seedling emergence because of water uptake improvement^9^. Treatment of tomato seeds with a DBD reactor increased the tomato yield. The bloom times, the height, the caulis, the extent of the plants, and the average weight, length, and diameter of each fruit in seven treatment groups from 4760 to 6800 V were increased distinctly^10^.

Exposing safflower seeds to low-pressure radio frequency (RF) 20 W argon gas discharge under two different constant pressures (1.6 and 16 Pa) improved the seed germination by 50.0%^6^. Treating soybean seeds with 80 W of low-pressure RF plasma at 15 s increased the germination and vigor indices by 14.7% and 63.3%, respectively^11^. *Arabidopsis thaliana* seeds treated with a plasma produced by DBD at 7.96 kV improved germination by 56.00%^12^. Cold plasma treatment affects physiological processes in plants, resulting in the promotion of seed germination and seedling growth^11,13^, increasing photosynthesis rate^14–16^, carbon and nitrogen metabolism^8,17^.

Waste biorefinery is an eco-friendly solution to produce fertilizers, fuels, and value-added products^18^. Waste biorefinery fertilizers have shown to increase the number of microorganisms in the soil^2^, which are essential in the carbon (C) and nitrogen (N) cycles and the bio-degradation of environmental contaminants^19–21^. Also, soil microbial biomass is essential for soil fertility^22^.

The microbial biomass in the soil is affected by several factors, particularly by the content of soil organic matter^23,24^. Pyroligneous acid (PA) is a waste bio-refinery product. It is a brown-colored liquid produced through gas condensation from burning waste plants and wood under limited oxygen conditions^25^. Pyroligneous acid contains over 200 chemical components, including acetic acid, hydroxy aldehydes, hydroxyl ketones, sugars, carboxylic acid, and phenolic acid^26,27^. Pyroligneous acid is an excellent source of organic ingredients^28^, which is non-toxic for humans, animals, plants, and the environment^29,30^. Pyroligneous acid has been used in traditional Japanese agriculture for more than 400 years^31^ to improve the quantity and quality of some agricultural products such as *Oryza sativa* L. (rice)*, Ipomoea batatas* L. (sweet potato)*, Saccharum officinarum* L. (sugarcane), and *Cucumis melo* L. (melon)^32–34^.

Biochar is a substance produced by a pyrolysis process at a low carbonization temperature (350–700 °C) under conditions of complete or partial anoxia. The carbon content of biochar is high, and its chemical properties are stable^35^. Adding biochar to the soil can counteract global climate change by sequestering carbon into the soil^36^. Many studies have investigated the positive influence of biochar. Biochar improves soil fertility, soil structure, texture, particle size distribution, and ultimately, plant growth^37^. Biochar application at a rate of 15 and 20 t ha^−1^ has significantly increased wheat and maize grain yield ^38,39^.

Azolla (*Azolla filiculoides* Lam.) is considered an invasive plant in wetlands, freshwater lakes, and ditches. It has the possibility to fix atmospheric nitrogen with asymbiotic cyanobacteria (i.e., *Anabaena azollae*)^40^. Azolla is suitable as compost due to its high nitrogen content that can enhance crop productivity^41–43^. Azolla compost is a natural fertilizer and can be mixed with rice straw^44^. It is a nitrogen source for plant nutrition, increasing plant yield^45^.

We aimed to increase the quality and quantity of winter wheat by treating the seeds with cold plasma before sowing and sowing the seeds in soil with added biorefinery waste products and azolla compost. Furthermore, we studied how these waste products affected the chemical and biological properties of the soil.

## Methods

### Experimental design and general methodology

The research was carried out in a research field at the Agricultural Research Station of Tarbiat Modares University, Tehran, Iran (35°41' N, 51°10' E, 1265 m above sea level) on sandy-loam soil during October 2016 and 2017. The area is semi-arid (according to the Köppen climate classification)^46^ characterized by warm and dry summers, long-term (30 years) mean annual rainfall of 232.6 mm, and a mean temperature of 17.6 °C, respectively. The experiment was conducted in a randomized complete block design, with four replications in two cropping seasons. The wheat was a landrace (Pishgam) from the central plateau of Iran. On 9 October 2016 and 9 October 2017, 140 kg ha^−1^ seeds were sown. Textures and soil elements were determined before sowing. The field was fertilized with nitrogen (150 kg ha^−1^), phosphorous (180 kg ha^−1^), and potassium (120 kg ha^−1^). Wheat seeds were sown manually at a depth of two inches in each plot. The area of the field was 1114 m^2^. Each plot was five meter wide and six meter long, including 20 rows. Row distance was 25 cm (400 plants ha^−1^).

Treatments included 1) seed priming by cold plasma, 2) soil application with pyroligneous acid, 3) a combination of cold plasma treatment and pyroligneous acid application, 4) biochar application, 5) a combination of cold plasma treatment and biochar application, 6) azolla compost application, and 7) a combination of cold plasma treatment and azolla compost application. Cold plasma treatment of seeds lasted 180 s. Pyroligneous acid (0.04 L m^−2^), biochar (0.5 kg m^−2^), and azolla compost (0.7 kg m^−2^) were incorporated into the soil before planting. Untreated seeds and soil were used as control.

#### Plasma treatment

**Figure 1:**
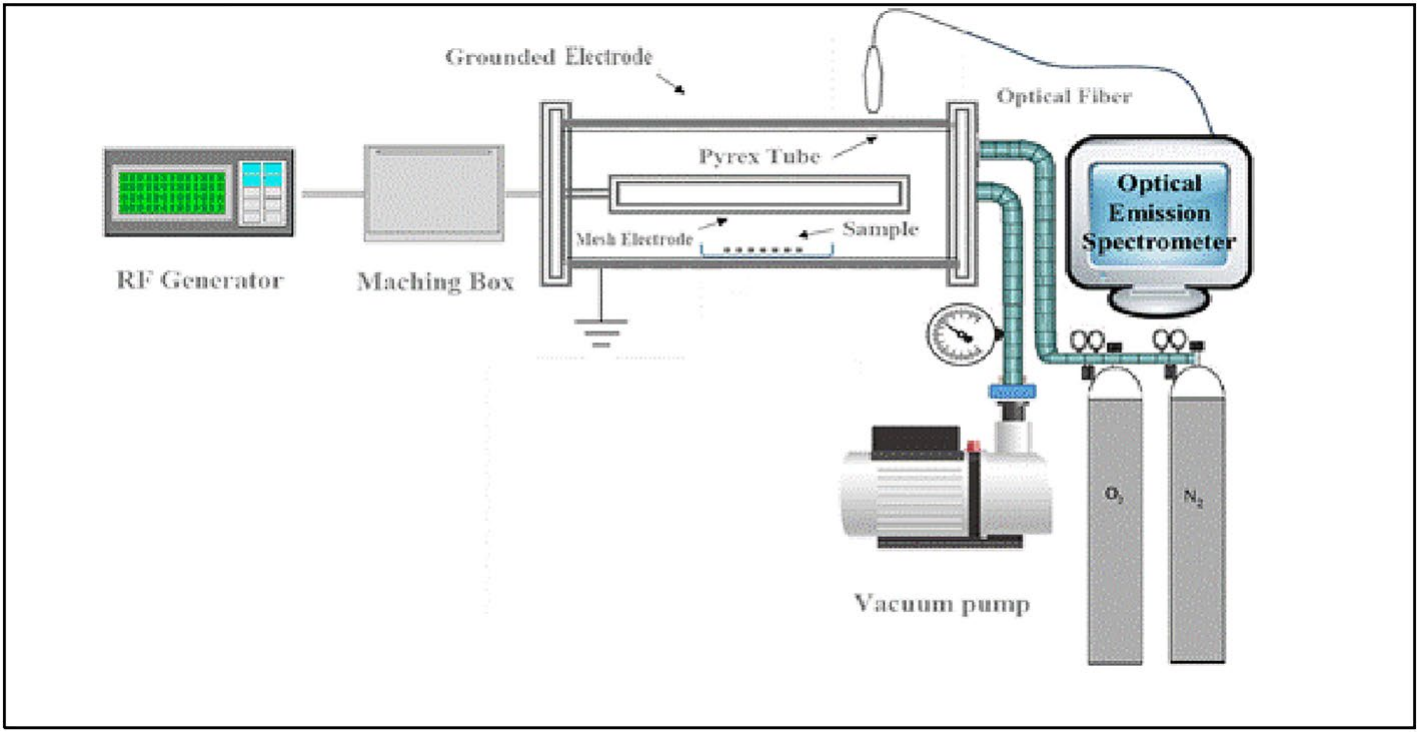
Process of treating wheat seeds by the RF plasma device.

Seed priming with plasma was applied using a radio frequency (RF) plasma reactor (manufactured by H.G and M.S, Shahid Beheshti University, Tehran, Iran) operated with air adjusted to 13.56 MHz, 50 W for 180 s. The vacuum chamber was made of a cylindrical Pyrex tube with an inner diameter of 80 mm and 300 mm. A metallic mesh grounded the outside of the Pyrex tube. The aluminum power electrode was fixed at the center of the cylinder (50 mm in width and 100 mm in length). The sample was placed under the Pyrex tube. The gap between the power electrode and the sample was 40 mm (Fig. 1). The seeds were exposed to a plasma flow for 180 s before sowing. The optical spectra was measured with an Optical Emission Spectroscopy (OES) Model HR2000 + ES with a wavelength range of 190 nm‒1.1 μm and an optical resolution of 0.9 nm (Ocean Insight Company, 3500 Quadrangle Blvd, Orlando, FL 32,817, USA).

**Figure 2:**
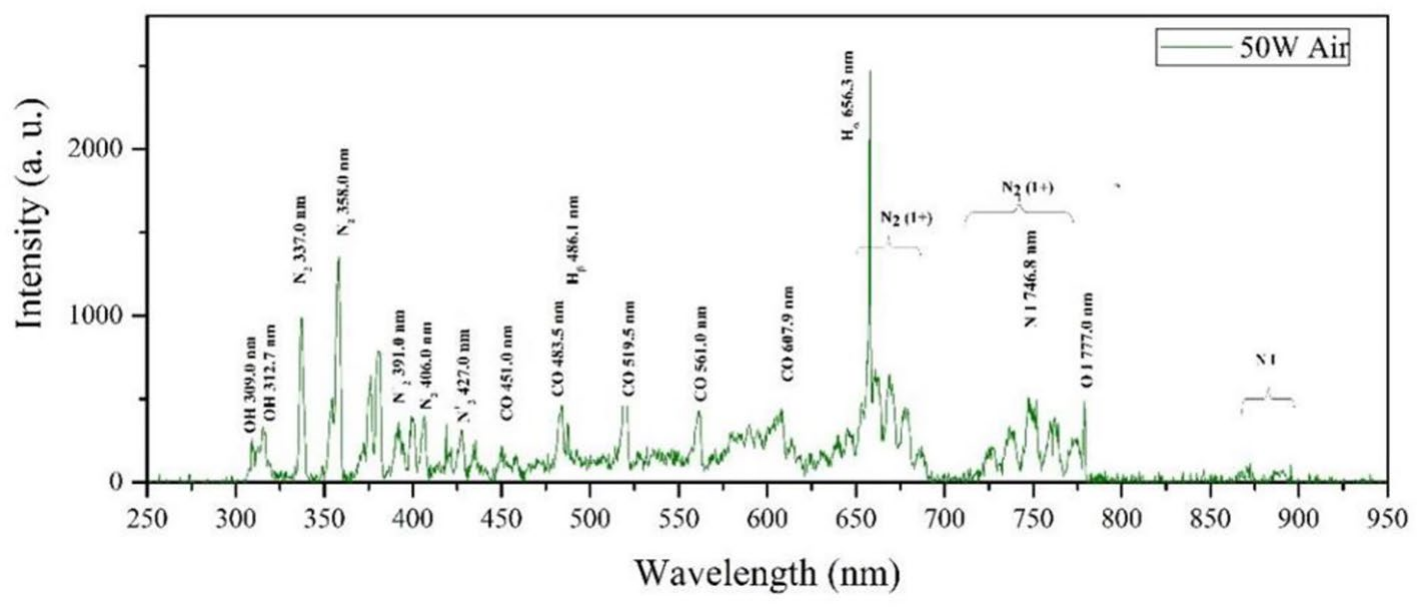
Optical spectra emitted from RF plasma for powers 50 W.

Figure 2 shows the optical spectra as a result of the RF plasma treatment. The main peaks are monitored in a range of 450−650 nm in the spectra, consisting of CO and hydrogen species. CO emission lines occur at 451.0, 483.5, 519.5, 561.0, and 607.9 nm, and an H emission line at 656.3 nm may represent products of the seed surface erosion process.

#### Pyroligneous acid, biochar, and azolla compost production

pyroligneous acid and biochar were produced by the carbonization process of lemon waste wood collected from the subtropical Mazandaran Province. At the beginning of the process, dry woods were placed in the closed furnace avoiding oxygen supply and heated to 350−700 °C. During the burning, the smoke from the wood went through a condenser and condensed to liquid (as pyroligneous acid). The solid part was obtained at 400 °C as biochar.

#### Measurements

Samples were taken from the middle of the plots to avoid border effects influencing the estimation of the grain yield and yield components. Photosynthesis (µmol m^−2^ s^−1^), intercellular CO_2_ concentration, and stomatal conductance (µmol m^−2^ s^−1^) were measured on the flag leaf 14 days after pollination by using a photosynthesis meter (model LI-COR 6400XT Version 6, Company, Lincoln, Nebraska, USA). Samples from green leaves in the middle of the canopy were taken fourteen days after the flowering stage to analyze the photosynthetic pigments.

#### Determination of chlorophyll and carotenoids

The content of chlorophyll and carotenoids was measured according to the method described by Krizek et al.^47,48^. Fourteen days after the flowering stage, four fresh leaf samples (0.2 g) were prepared. The samples were extracted in 10 ml 80% acetone solution and centrifuged for 10 min at 1600 rpm. The absorption spectra were measured at 663, 645, and 470 nm with a spectrophotometer (Model Perkin Elmer 3110, Granite Quarry, NC, USA).

#### Determination of anthocyanin concentration

We extracted 0.2 g fresh leaves in 15 ml glass centrifuge tubes containing 10 ml of acidified methanol (methanol: HCl, 99:1, vol: vol) and kept the tubes in the dark overnight. The absorbance was measured at 550 nm. The anthocyanin concentration was calculated using an extinction coefficient of 33,000 mol^−1^ cm^−1^^48^.

#### Determination of protein content in wheat grain

Kjeldahl method was used to measure protein content^49^. Approximately 1 g of raw material was hydrolyzed with 15 ml concentrated sulfuric acid (H_2_SO_4_) containing two copper catalyst tablets in a heat block (Kjeltec system 2020 digestor, Tecator Inc. Herndon, VA, USA) at 420℃ for two hours. After cooling, H_2_O was added before neutralization and titration. The amount of total nitrogen in the raw materials was multiplied with both the traditional conversion factor of 6.25 and the species-specific conversion factors to determine the total protein content.

#### Determination of starch content in wheat grains

The starch content was estimated as described by McCleary et al. (1994)^50^ using the megazyme total starch analysis (AA/AMG) procedure. First, 100 mg grain was milled in an UDY cyclone mill to pass through a 0.5 mm screen, wetted with 0.2 ml ethanol, and treated with thermostable α-amylase (AA) to partially hydrolyze the starch. After completely dissolving the starch, dextrin was quantitatively hydrolyzed to glucose by amyloglucosidase (AMG). The starch content was estimated based on the glucose measurements.

#### Determination of the microbial biomass carbon (C_mic_) and nitrogen (N_mic_) in the soil

Microbial biomass carbon (C_mic_) and nitrogen (N_mic_) were determined by the chloroform fumigation–extraction method^51^. C_mic_ was measured by using a 15 g oven-dry equivalent of the field-moist soil sample, and the C content was extracted using the chloroform-fumigation-extraction method (FE) described by Vance et al. (1987)^52^. The concentration of C_org_ in the extractant was determined by a carbon analyzer (SHI-MADZU Model TOC-5050, Milton Freewater, Oregon, USA) after acidification with one drop of 2 M HCl to remove any dissolved carbonate. Carbon biomass was calculated as follows: C_mic_ = EC/*K*_EC_ where EC = (organic C extracted from fumigated soil)—(organic C extracted from non-fumigated soil) and *K*_EC_ = 0.45, which is the proportionality factor to convert EC to C_mic_^53^.

Microbial biomass nitrogen (N_mic_) was determined from the total nitrogen (N_total_) released during fumigation–extraction^54^. Soil N_mic_ was determined by the chloroform-fumigation-incubation method (FI) as described by Horwath and Paul (1994)^51^ using a 15 g oven-dry equivalent of the field-moist soil sample after adjusting the moisture content to 55% of the water-holding capacity (WHC). Samples were conditioned for seven days at 25℃. The NH_4_^+^-N in the extracts was determined in 20 ml aliquots by steam distillation^55^. Biomass N was calculated using the equation: N_mic_ = EN/*k*_IN_ Where E_N_ = (flush of NH_4_^+^-N due to fumigation)–(NH_4_^+^-N produced in the non-fumigated soil during ten days incubation) and *k*_IN_ = 0.57, which is the proportionality factor to convert EN to N_mic_^56^. The C_mic_ and N_mic_ values were determined on the < 2 mm mesh field-moist samples. All results reported are averages of duplicated analyses and are expressed on a moisture-free basis. Moisture was determined after drying at 105 °C for 48 h.

#### Statistical analysis

Years were analyzed separately because Bartlett’s test was significant for most traits measured. The effect of treatments was determined by analysis of variance (ANOVA) using SAS (Version 9.2) (SAS, 2002) software^57^. The assumptions of variance analysis were tested by ensuring that the residuals were random, homogenous, with a normal distribution about/above a mean of zero. LSD test at the 0.01 probability level was used to check significant differences between means.

## Results

### Effect of combination of cold plasma and azolla compost on wheat treats

**Figure 3:**
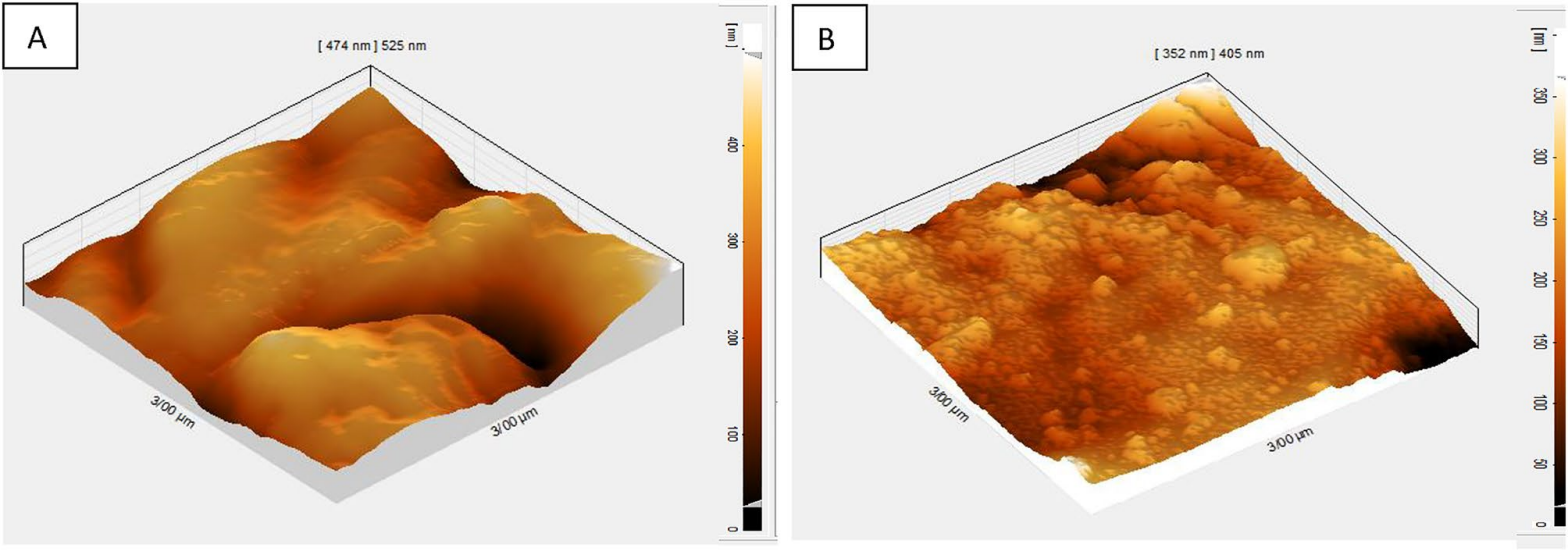
Atomic force microscope (AFM) images of seed surface at 10 µm. (**A**) control (without plasma treatment). (**B**) seed surface after receiving 180 s, 50 W plasma treatment. The images was taken from identical locations on the dorsal side of the wheat kernels.

**Figure 4:**
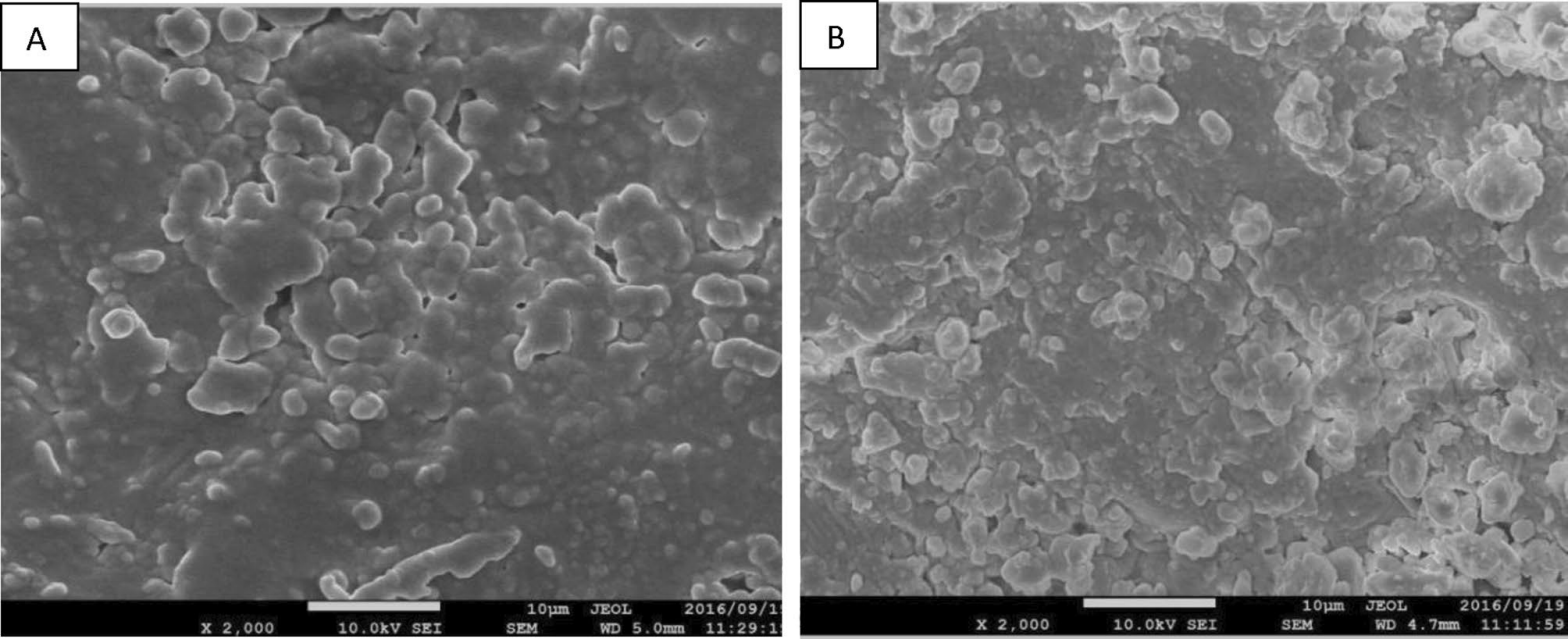
Scanning electron microscope (SEM) images of seed surface at 10 µm. (**A**) control (without plasma treatment). (**B**) seed surface after receiving 180 s, 50 W plasma treatment. The images were taken from identical locations on the dorsal side of the wheat kernels.

By examining plasma light emission spectroscopy with Scanning Electron Microscopy (SEM) and Atomic Force Microscopy (AFM) images of the sample surface, it has been shown how plasma treatment can increase seed growth components. Atomic Force Microscopy image was taken from two samples of wheat treated with air plasma at 50 W for 180 s. Atomic force microscope images showed the roughness and morphology of the seed surface of the two wheat seed samples (Fig. 3). The figure on the left shows the control sample. No carvings were observed on the seed surface of the control sample (Fig. 3). Figure 4 shows Scanning Electron Microscope (SEM) images of two wheat seed samples. The figure on the left shows a sample of a 50 W 180 s air plasma treatment. The bombardment of ions and free radicals in plasma is well seen on the sample surface, which has resulted in seed hydrophilicity. However, an excessive bombardment of the seed and rising temperature may cause the sample to die, which was reflected in the seed germination percentage.

The plasma treatment effectively removed a thin layer of the seed lipid coat by reducing the bio-polymeric chains' length and the average molecular weight on the seed surface (Figs. 3 and 4). Table 1 shows that years (Y) and treatments (T) significantly affected the biochemical content in the wheat grains at a 1% level. There was also a significant interaction effect (Y*T). In both years, the combination of plasma treatment with azolla compost and pyroligneous acid treatments significantly increased the anthocyanin content in the leaves compared with the control. The highest anthocyanin content was obtained when the plasma treatment was combined with azolla compost. This treatment increased the anthocyanin by 39.9% compared with the control (Table 2).

**Table 1:** Analysis of variance showing the effect of year (Y), treatments (T) (application of plasma, pyroligneous acid, azolla compost and biochar) and interactions (T*Y) of on the biochemical responses of wheat.

Source	df	Mean square			
		Anthocyanin	Carotenoid	Total chlorophyll	Net photosynthesis rate
Y	1	19.90**	12.25**	43.85**	231.14**
R(Y)	4	0.02^ ns^	0.37^ ns^	0.43^ ns^	1.20^ ns^
T	7	5.07**	3.67**	1.88**	10.20**
T*Y	7	1.25*	0.53^ ns^	0.54^ ns^	5.82**
Error	27	0.81	0.52	0.36	1.33
CV (%)		15.28	14.88	15.24	14.76

*p < 0.05, **p < 0.01, Y, year; R(Y), replication year; T, treatments; T*Y, interaction between treatments and years; df, degree of freedom; CV, coefficient of variation.

**Table 2:** Mean comparison of effect of application of plasma, pyroligneous acid, azolla compost and biochar on biochemical responses of wheat.

Traits								
Treatments	Anthocyanin (µmol. ml^−1^)		Carotenoid (µmol. ml^−1^)		Total chlorophyll (µmol. ml^−1^)		Net photosynthesis rate (µmol. ml^−1^)	
	2016	2017	2016	2017	2016	2017	2016	2017
Plasma	5.59 ± 0.25d-e	6.39 ± 0.69bc	5.38 ± 0.21 cd	5.44 ± 0.20bc	3.31 ± 0.41bc	4.56 ± 0.21 cd	6.02 ± 0.67 cd	7.23 ± bc
Pyroligneous acid	5.68 ± 0.37c-e	5.94 ± 0.47cde	5.27 ± 0.29d	4.66 ± 0.88c	3.22 ± 0.24bc	4.56 ± 0.51 cd	5.31 ± 0.33d	7.04 ± 0.51d
Plasma + Pyroligneous acid	6.13 ± 0.16ab	6.44 ± 0.78b	5.67 ± 0.30bc	6.60 ± 0.96a	3.60 ± 0.48a	5.15 ± 0.05a	7.87 ± 0.57a	7.03 ± 0.08a
Azolla compost	5.13 ± 0.76de	5.93 ± 0.81de	4.97 ± 0.21e	4.41 ± 0.83d	3.21 ± 0.51cde	4.21 ± 0.13e	5.11 ± 0.73de	6.97 ± 0.87de
Plasma + Azolla compost	6.23 ± 0.17a	6.56 ± 0.76a	6.01 ± 0.42a	5.72 ± 0.41a	3.38 ± 0.07bc	4.71 ± 0.38bc	6.13 ± 0.35c	7.34 ± 0.21bc
Biochar	4.29 ± 0.51e-f	5.68 ± 0.78e	4.63 ± 0.29ef	4.25 ± 0.18de	2.80 ± 0.27e	4.07 ± 0.21ef	4.54 ± 0.75e	6.94 ± 0.14de
Plasma + Biochar	5.97 ± 0.58bc	5.72 ± 0.46b	5.74 ± 0.22b	5.68 ± 0.16ab	3.48 ± 0.91b	4.91 ± 0.26b	7.66 ± 0.42b	7.33 ± 0.33ab
Control	4.12 ± 0.88f.	5.02 ± 0.35f.	4.23f. ± 0.57 g	4.05 ± 0.25e	2.68 ± 0.18ef	3.23 ± 0.28f.	4.07 ± 0.41f.	6.64 ± 0.12e

Means in each column followed by similar letter(s) are not significantly different at 5% probability level using LSD Range Test.

In the second year, the combination of plasma treatment and azolla compost, biochar, and pyroligneous acid significantly increased the carotenoid content compared with control. In both years, the combination of plasma treatment and azolla compost increased the carotenoid content most (Table 2).

**Figure 5:**
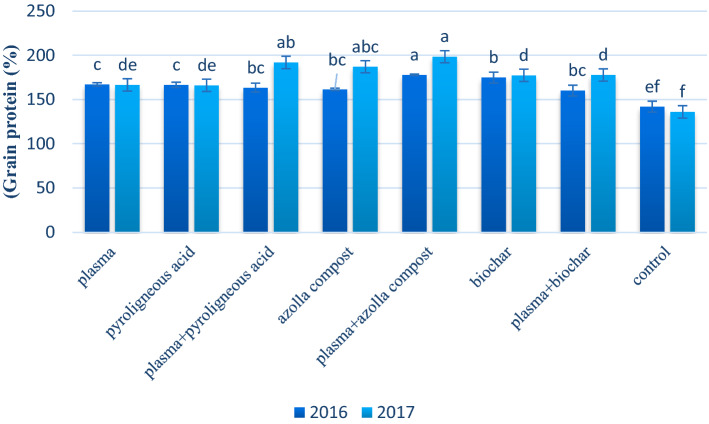
Mean comparison of effect of application of plasma, pyroligneous acid, azolla compost and biochar on grain protein of wheat.

In both years, cold plasma treatment combined with azolla compost application significantly increased the grain protein by 35.0% compared with the control (Fig. 5). In the second year, plasma treatment combined with azolla compost, and plasma treatment combined with pyroligneous acid significantly increased the amount of grain protein.

### The effect of combining cold plasma and pyroligneous acid treatment

The combination of plasma and pyroligneous acid significantly increased the total chlorophyll content and net photosynthesis rate in both years (Table 2). There was a significant difference between years at a 1% level in intercellular CO_2_ concentration and stomatal conductance. In addition, there was a significant difference between treatments and interaction between treatments and years on mesophyll conductance and stomatal conductance of wheat at a 1% level (Table 3).

**Table 3:** Analysis of variance of application of plasma, pyroligneous acid, azolla compost and biochar on physiological responses of wheat.

Source	df	Mean square		
		Intercellular CO_2_ concentration	Mesophyll conductance	Stomatal conductance
Y	1	130,656.94**	1117.97	0.00443**
R(Y)	4	2836.81*	3299.07	0.00009**
T	7	2388.97*	8869.49**	0.0002**
T*Y	7	1626.19	4547.52**	0.0001**
Error	27	1369.21	1667.37	0.0001
C.V. (%)		16.38	18.09	12.10

*p < 0.05, **p < 0.01, Y, year; R(Y), replication year; T, treatments; T*Y, interaction between treatments and years; df, degree of freedom; C.V., coefficient of variation.

The combination of plasma and pyroligneous acid and azolla compost increased the intercellular CO_2_ concentration in the first year (Table 4). The highest intercellular CO_2_ concentration was observed in both years when plasma and pyroligneous acid treatments were combined (Table 4).

**Table 4:** Mean comparison of effect of application of plasma, pyroligneous acid, azolla compost and biochar on physiological responses of wheat.

Traits						
Treatments	Intercellular CO_2_ concentration (mol m^−2^ s^−1^)		Mesophyll conductance (mol m^−2^ s^−1^)		Stomatal conductance (mol m^−2^ s^−1^)	
	2016	2017	2016	2017	2016	2017
Plasma	0.029 ± 0.09c	0.041 ± 0.05a-c	294 ± 1.21a	225.33 ± 1.21bcd	196.13 ± 4.25abc	260.44 ± 1.69 cd
Pyroligneous acid	0.035 ± 0.02a	0.052 ± 0.07a	206.67 ± 0.28e	254 ± 0.88ab	189.1 ± 6.37c	263.2 ± 2.47ab
Plasma + Pyroligneous acid	0.031 ± 0.05b	0.045 ± 0.06ab	291 ± 1.31ab	244 ± 1.96b	205.06 ± 4.06a	305.33 ± 1.78a
Azolla compost	0.026 ± 0.08de	0.036 ± 0.06 cd	198 ± 0.21ef	182.33 ± 0.88f.	164.28 ± 5.76ef	231.68 ± 4.81d
Plasma + Azolla compost	0.028 ± 0.05 cd	0.045 ± 0.07ab	213.67 ± 0.42 cd	198.33 ± 1.41d	198.05 ± 4.07ab	262.37 ± 4.76bc
Biochar	0.025 ± 0.06e	0.037 ± 0.05 cd	233 ± 0.29c	257 ± 0.12a	173.97 ± 4.51d	209.97 ± 4.78e
Plasma + Biochar	0.027 ± 0.08d	0.039 ± 0.08c	163.67 ± 1.22 g	221.67 ± 2.18bcd	183.84 ± 4.58 cd	232.53 ± 4.46d
Control	0.025 ± 0.06ef	0.038 ± 0.07 cd	147.46 ± 0.57 h	195 ± 2.16de	169.55 ± 5.88e	209.09 ± 3.35e

Means in each column followed by similar letter(s) are not significantly different at 5% probability level using LSD Rang Test.

**Figure 6:**
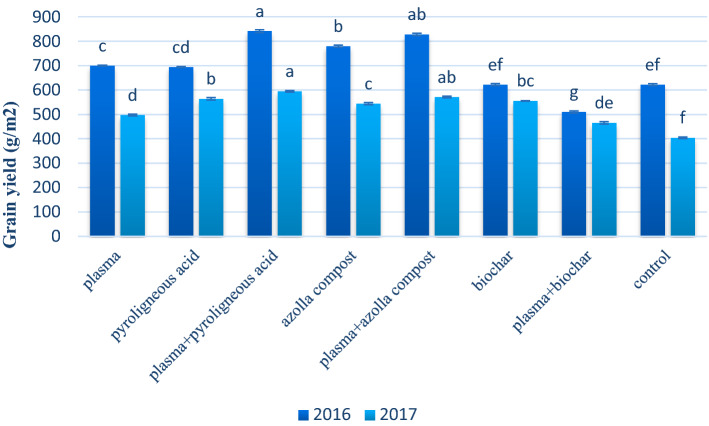
Mean comparison of effect of application of plasma, pyroligneous acid, azolla compost and biochar on grain yield of wheat.

There was a significant difference between years and interaction between years and treatments at a 1% level according to LSD test for wheat grain yield (Table 5). In both years, however, their combination of plasma and pyroligneous acid and azolla compost increased the grain yield (Fig. 6). The highest grain yield was observed when plasma and pyroligneous acid were combined.

**Table 5:** Analysis of variance of application of plasma, pyroligneous acid, azolla compost and biochar on yield, protein and starch content of wheat in the field.

Source	df	Mean square		
		Grain yield	Grain starch	Grain protein
Y	1	497,297.05**	10,364.44**	40.08**
R(Y)	4	62,637.13*	1481.12*	1.57
T	7	521,938	2183.41**	2.17**
T*Y	7	341,475.58**	770.88	2.81**
Error	27	6483.66	564.01	0.81
C.V. (%)		13.95	14.81	7.98

* and ** Significant at 5% and 1% probability levels, respectively. Y, year; R(Y), replication year; T, treatments; T*Y, interaction between treatments and years; df, degree of freedom; C.V., coefficient of variation.

### Effect of pyroligneous acid on wheat treats

In 2017, cold plasma combined with pyroligneous acid and cold plasma combined with azolla compost and pyroligneous acid significantly increased stomatal conductance (Table 4). In both years, the maximum stomatal conductance (38.0% increase) was obtained in the pyroligneous acid application (Table 4).

The plasma treatment and plasma treatment combined with pyroligneous acid significantly increased the mesophyll conductance in the first year. In the second year, biochar and pyroligneous acid treatments significantly increased the mesophyll conductance (Table 4).

### Effect of biochar on wheat treats

There was a significant difference between years and treatments at a 1% level according to LSD test for wheat grain starch (Table 5).

**Figure 7:**
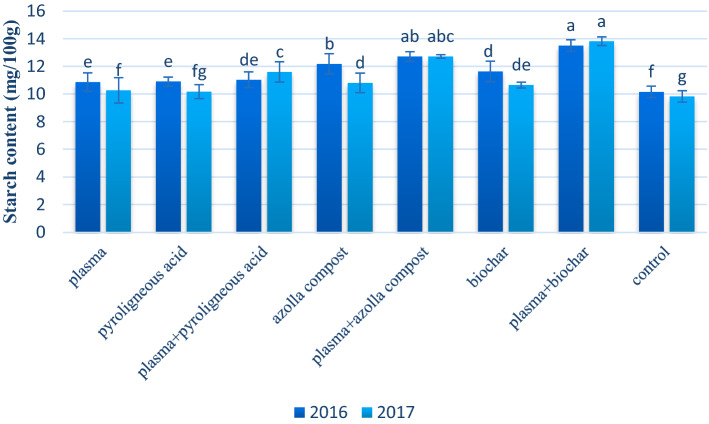
Mean comparison of effect of application of plasma, pyroligneous acid, azolla compost and biochar on starch content of wheat.

Plasma seed priming combined with azolla compost and biochar significantly increased the starch content in both years. The highest amount of starch (36.0% increase) was achieved by combining plasma seed priming with biochar (Fig. 7).

### Effect of different treatments on the content of chemicals in the soil

The application of pyroligneous acid and the combination of pyroligneous acid and plasma seed priming significantly increased available phosphor, zinc, iron, and calcium in the soil (Table 6). Application of Azolla compost to the soil and the combination of plasma seed priming with azolla compost also significantly increased the amount of nitrogen in the soil. When biochar and plasma seed priming were combined, the amount of organic carbon significantly increased compared with the controls (Table 6).

**Table 6:** Mean comparison of effect of application of plasma, pyroligneous acid, azolla compost and biochar on chemical changes of soil in the field average 2016–17.

Treatments	Organic carbon	N	Available P	Available K	Available Zn	Available Fe	Available Ca
	OC%	(%)	(ppm)	(ppm)	(ppm)	(ppm)	(ppm)
Plasma	0.896f.	0.133e	32e	276 fg	1.92d	3.12c	2386 fg
Pyroligneous acid	0.903e	0.139de	40a	349a	3.03ab	3.74ab	2688a
Plasma + Pyroligneous acid	0.919d	0.140d	39ab	347ab	3.08a	3.76a	2686ab
Azolla compost	0.980cd	0.171ab	35c	316c	2.84b	3.18d	2348ef
Plasma + Azolla compost	0.984c	0.176a	34cd	314cd	2.81bc	3.24e	2368e
Biochar	1.030ab	0.151c	35c	316c	2.31cd	3.58b	2448c
Plasma + Biochar	1.035a	0.151c	37b	313cde	2.32c	3.51ab	2432cd
Control	0.890 g	0.132ef	32e	277f.	1.91de	3.13ef	2390f.

Means in each column followed by similar letter(s) are not significantly different at 5% probability level using LSD Range Test.

All treatments where Azolla compost was included significantly increased the microbial biomass carbon content (up to 29.0%) compared with the control (Table 7). Also, the combination of biochar and plasma seed priming significantly increased the microbial biomass carbon (16.0%) (Table 7).

**Table 7:** Mean comparison of effect of application of plasma, pyroligneous acid, azolla compost and biochar on microbial changes of soil in the field average 2016–17.

Treatments	Microbial Biomass Carbon (mg/kg)	Microbial Biomass Nitrogen (mg/kg)
Plasma	132e	9.91e
Pyroligneous acid	141c	10.12d
Plasma + Pyroligneous acid	143cd	10.21d
Azolla compost	141c	12.78a
Plasma + Azolla compost	140d	12.76a
Biochar	150ab	11.56bc
Plasma + Biochar	152a	11.67b
Control	131ef	9.88ef

Means in each column followed by similar letter(s) are not significantly different at 5% probability level using LSD Range Test.

## Discussion

Cold plasma treatment positively affected the physiological and biochemical measured parameters and yield (Tables 2 and 4). A positive effect of cold plasma on photosynthesis rate has been reported previosly^58–60^. The increasing germination caused by the plasma treatment is probably due to 1) changes in the surface of the seed coat resulting in increasing water and nutrient uptake, 2) changes that occur inside the cell that increases the biochemical activities^14,61^. When the plasma treatment enhances water and nutrient uptake, it results in greater seed weight^62,63^. This effect of plasma treatment has also been shown for beans (*Phaseolus vulgaris* L.)^64^.

Several OH and nitrogen species peaks can also be observed in the 250–400 nm spectral range after plasma treatment. Spectral emissions at 283.4, 309.0, and 312.7 nm indicate the presence of OH species, and a peak at 357.0 nm also corresponds to molecular nitrogen in the RF plasma. Plasma interaction with material surfaces leads to chemical changes on the surface, as a result increasing seed surface hydrophilicity characteristics.

The oxygen gas spectrum, showed two basic peaks, 777.0 nm, and 844.0 nm, which correspond to the first excited state of the oxygen atom (Fig. 2). In low-pressure cold plasma, free electrons' activation and excitation of molecules occur. For example, in air plasma, which is a combination of nitrogen and oxygen, the following processes also happen:

$$\rm{ O \, + \, N_{2} \to \, NO \, + \, N }$$

$$\rm{N \, + \, O_{2} \to \, O \, + \, NO}$$

$$\rm{N_{2} + \, e \, \to \, N*_{2}}$$

$$\rm{N*_{2} + \, O2 \, \to \, 2O \, + \, N_{2}}$$

In other words, chain processes lead to the formation of unstable molecules such as ozone and nitrous oxide. The last reaction results from the quasi-stable-neutral collision of the nitrogen and oxygen molecules. This reaction, together with active compounds such as O, O_2_, O_3_, and the collision of ions and free radicals with the seed coat, probably has the most significant effect on surface carving and activation^64^.

The chemical interaction of active compounds leads to the formation of hydrophilic chemical bonds on its surface, changing the surface properties from a non-polar state (hydrocarbons and fats) to polar bonds, including oxygen, thus increasing the hydrophilicity of the seed surface. In the 600−4000 cm^−1^, peaks related to O–H and C−O bands belonging to the functional group of phenols, C−O carbonyl esters, and C−N and N–H functional groups of amines were observed. Therefore, seed hydrophilicity increases on the seed surface by breaking carbon bonds (often due to the presence of lipid on the seed surface) and the penetration of polar agents, including oxygen and nitrogen atoms that are actively present in the plasma.

The combination of pyroligneous acid and cold plasma treatment significantly affected the total chlorophyll, intercellular CO_2_ concentration, and net photosynthesis rate of winter wheat compared to the control (Tables 2 and 4). Previous studies have shown the positive effect of pyroligneous acid on accelerating somatic embryogenesis^65^, enhancing seed germination^66^, increasing flowering rate, and root biomass^67–69^. It has also been shown that pyroligneous acid can increase the growth of roots, stems, tubers, leaves, flowers, and fruits and improves soil fertility^70^.

Pyroligneous acid comprises ester compounds^70^. The esters can increase the chlorophyll content and stimulate the photosynthesis in several plants, increasing the synthesis of carbohydrates and amino acids in plants and, consequently, increasing plant yield and making plants more resistant to pests and diseases^71,72^. The biophysical properties of esters include lipid solubility. Transmembrane permeability may be a critical factor in the bioactivity of esters in the plant cells' physiological processes. The esters provoke stomatal closure in several plant species belonging to *Solanaceae*, *Leguminosae*, *Brassicaceae*, and *Gramineae* due to increasing CO_2_ uptake in plant leaves^73^. The stomatal aperture regulates CO_2_ uptake and water transpiration and influences the plant's resilience under drought stress condition^74^. Also, the accumulation of various metabolites, such as intermediates of the Calvin cycle, paramylon precursors, and pyruvate, as a substrate for pyruvate: NADP + oxidoreductase, by enhancements in photosynthetic capacity, is one of the key metabolic factors for the role of esters^75^.

Increase in stomatal conductance results in enhancement of the CO_2_ uptake, which increases the photosynthesis rate, and the flow of supply materials^76^ for grain performance^77,78^. The highest grain yield was achieved by combining cold plasma treatment with the application of pyroligneous acid in both years (Fig. 6).

Pyroligneous acid contains 15 macro and microelements, including calcium, cadmium, chromium, copper, iron, potassium, manganese, aluminum, sodium, and zinc^72^. It contains more than 200 substances, including organic acid (e.g., formic acid, acetic acid, acetic acid, propionic acid, butyric acid, phenol group, carbonyl group, formaldehyde, acetaldehyde, ethanol, methanol, acetyl)^79,80^. The simultaneous presence of acetic acid and iron and calcium cations creates a complex where the ionic bond replaces the covalent bonds, preventing iron deposition in the soil and leaching other elements^72^. Besides, pyroligneous acid helps enrich the phosphorus, calcium, iron, and potassium contents in the soil (Table 6).

The plasma treatment combined with azolla compost application increased the protein content (Fig. 5). Organic fertilizers such as azolla compost are known as a source of nitrogen. Also, it has been shown that compost increases nitrogen absorption in the plant^81^. High nitrogen absorption leads to an increased amount of protein content in the grain of crop^72^. The maximum concentration of anthocyanin in cold plasma treatment and azolla compost seems to be conditioned by the availability of assimilates^82,83^. In this way, nitrogen enters the microbial parts to help extend their activity. Compost feedstock can affect the carbon and nitrogen cycling dynamics of the soil^84^. Azolla compost can effectively increase soil organic matter content, resulting in a higher carbon and nitrogen mineralization^85^ and increased microbial biomass^86^. Compost affects the soil fertility gradients by C and N availability and, subsequently, improves soil microbial activity^87^.

Biochar application increased the starch content significantly (Fig. 7). Biochar was obtained by decomposing the forest, residual plants, and manure residues containing essential nutrients elements such as carbon, nitrogen, and sulfur^88^. Numerous studies have shown that biochar can significantly increase nutrient uptake, increasing starch content^89–91^. In addition, biochar also retains soil nutrients and soils cation exchange capacity due to less leaching^92–94^.

Azolla compost and biochar increased the microbial biomass nitrogen (MBN) and microbial biomass carbon (MBC). The flexibility of MBC is probably due to microbial death or the release of intracellular substrates^95^. Increasing carbon input (through organic amendment application) can increase the soil organic matter degradation rate^96^ as microbes use labile C to decompose recalcitrant soil organic matter^97^.

Biochar in soil has several direct and indirect influences on soil biota because of changes in several abiotic factors, including soil pH or altered substrate quality as a source of energy^98^. In addition, biochar increases positive changes to soil characteristics of water contents, EC, and pH status; all of these factors, directly and indirectly, affect the activity of life in the soil and, as a result, increase soil microbial activity^99^. Hence, soil microbial activities are related to soil fertility and agricultural productivity^100^.

## Conclusion

A combination of plasma seed priming and pyroligneous acid application increased the grain yield of the winter wheat. Furthermore, combining seed plasma treatment with biochar and azolla compost application increased the starch and protein content in the harvested grain. The biorefinery products also improved the quality of the soil significantly. The amendments enhanced the soil microbial biomass and improved essential soil factors affecting the root system's ability to uptake soil water and nutrients. Cold plasma seed priming seems to be a promising technique to improve seed germination performance, plant establishment, growth, and grain yield. This research showed ways to improve crop yield and contribute to preserving the environment by reducing the effects of climate change side-effects by reducing the need for agrochemicals (fertilizers) during the growth cycle and improving soil microbial biomass. By combining cold plasma treatment with pyroligneous acid, biochar, and azolla compost, we improved the soil as a growth medium and the performance of winter wheat significantly, resulting in a higher yield.

## Supplementary Information

Supplementary Information.
